# Comparative study of catalytic activities among transition metal-doped IrO_2_ nanoparticles

**DOI:** 10.1038/s41598-018-35116-w

**Published:** 2018-11-13

**Authors:** Hangil Lee, Joo Yeon Kim, Si Young Lee, Jung A. Hong, Namdong Kim, Jaeyoon Baik, Yun Jeong Hwang

**Affiliations:** 1 Department of Chemistry, Sookmyung Women’s University, Seoul, 140-742 Republic of Korea; 2 Clean Energy Research Center, Korea Institute of Science and Technology, Seoul, 02792 Republic of Korea; 3 Division of Energy and Environmental Technology, KIST School, Korea University of Science and Technology, Seoul, 02792 Republic of Korea; 4 Beamline Research Division, Pohang Accelerator Laboratory (PAL), Pohang, 790-784 Republic of Korea

## Abstract

Catalytic activities of transition metal-doped IrO_2_ nanoparticles (TM-IrO_2_ NPs; TM = Cr, Mn, Fe, Co, or Ni) are compared for various oxidation reactions such as electrochemical oxygen evolution reaction (OER), gas-phase photo-oxidation of thiol function group, and CO oxidative conversion. Here, we discovered a series of TM-IrO_2_ catalysts have a common activity trend for these oxidation reactions, and their activities are closely related with modified electronic states of IrO_2_, strongly affected by the types of the transition metal across the periodic table. For all oxidation reactions, Cr- and Mn-IrO_2_ achieved the highest oxidation catalytic activity, and sequentially decreased activities were obtained with Fe, Co, and Ni doped IrO_2_. For instance, the highest OER activity was achieved by Cr or Mn doping exhibiting the smallest overpotential η = 275~230 mV at 10 mA/cm^2^, while Ni-IrO_2_ showed rather larger overpotential (η = 347 mV) even compared with non-doped IrO_2_ (η = 314 mV). Scanning transmission X-ray microscopy and high-resolution photoemission spectra of TM-IrO_2_ indicated dopant metals modified the Ir-O interaction and thus increasing oxygen vacancy defects in IrO_2_. Strongly positive correlation was observed between the catalytic activities and vacancy states. The amount of defect related signals was observed the most for Cr- or Mn-IrO_2_, less so for Fe- or Co-IrO_2_, and unnoted for Ni-IrO_2_ compared with bare IrO_2_. Based on these catalytic activities and surface spectroscopic analysis results, vacancy defects induced by doping in TM-IrO_2_ NPs are proposed to contribute to enhance the oxidation activities.

## Introduction

Iridium oxide (IrO_2_) has been demonstrated as an active catalyst material in impressively wide ranges of applications for electrocatalyst^1–4^, catalyst^5–8^, photocatalyst, etc^9–11^. For instance, IrO_2_ has excellent electrocatalytic activities for oxygen evolution reaction (OER) in batteries or water-electrolysis and considered as a benchmarking material to compare the catalytic activities of others^12–14^. Defects or even amorphous structure on the IrO_2_ surface have been proposed as the active sites of electrochemical OER. In addition to the electrochemical oxidation reaction, IrO_2_ nanoparticles have been recently reported to display photocatalyst capability, in particular, to split water under UV irradiation through the excitation from the O-*p* band to the Ir-*d* band^15,16^.

Along with mechanistic studies of IrO_2_ catalyst^17,18^, attempts to enhance their catalytic activities have been tried via alloying or inserting dopants such as metals or anions into these nanoparticles (NPs)^19–23^. In IrO_2_, transition metal dopants can access various oxidation states which can disturb the bonding between Ir and O and consequentially modulate the electronic/chemical states of IrO_2_. Therefore, it can be hypothesized that these modified electronic states vary the intrinsic catalytic activity. Although the active sites of the IrO_2_ catalysts can be different depending on the catalytic reactions due to the different chemicals and reaction pathways, similar strategies such as doping or alloying have been successfully demonstrated to improve activity of IrO_2_. This can be valid if there is a related active factor of various reactions that controls catalytic activity on the IrO_2_ surfaces.

To understand the possible relation of the active species and catalytic activity of IrO_2_ for various reactions, we inserted various transition metal ions (TM^+^) into IrO_2_ NPs to enhance their own catalytic activities and then systematically compared the catalytic activities of these transition metal-doped IrO_2_ (TM-IrO_2_) NPs for different types of oxidation reactions. In particular, the cost-effective transition metals such as Cr, Mn, Fe, Co, and Ni were doped in IrO_2_ NPs by carrying out facile hydrothermal syntheses. Understanding the chemical/electronic structures of IrO_2_ NPs is important, but complexity in the electronic structures of the solid material can make it particularly difficult to determine the chemical state of the active surface species experimentally. Spectroscopic surface analysis techniques can provide the useful information to understand the surface states on IrO_2_ and modified IrO_2_ NP surface^24^. Therefore, the TM-IrO_2_ NPs were characterized by using scanning electron microscopy (SEM), Raman spectroscopy, and scanning transmission X-ray microscopy (STXM) and the influence of the dopant types on the electronic structures was studied. Then, the catalytic activities of the TM-IrO_2_ NPs were assessed for the OER in an aqueous solution by taking electrochemical measurements, for the photocatalytic oxidation of 2-aminothiophenol (2-ATP) in a gas phase media by performing high-resolution photoemission spectroscopy (HRPES), and for the thermal CO conversion to CO_2_ by measuring mass spectroscopy. We observed there is a common catalytic activity trend for all these different oxidation reactions. Espeically, highly enhanced catalytic activity was achieved when Cr or Mn was doped in IrO_2_ NPs in which modified electron states showed largest generation of vacancy defects proposing as a main contributor.

## Results and Discussion

### Characterization of transition metal doped IrO_2_ nanoparticles

**Figure 1:**
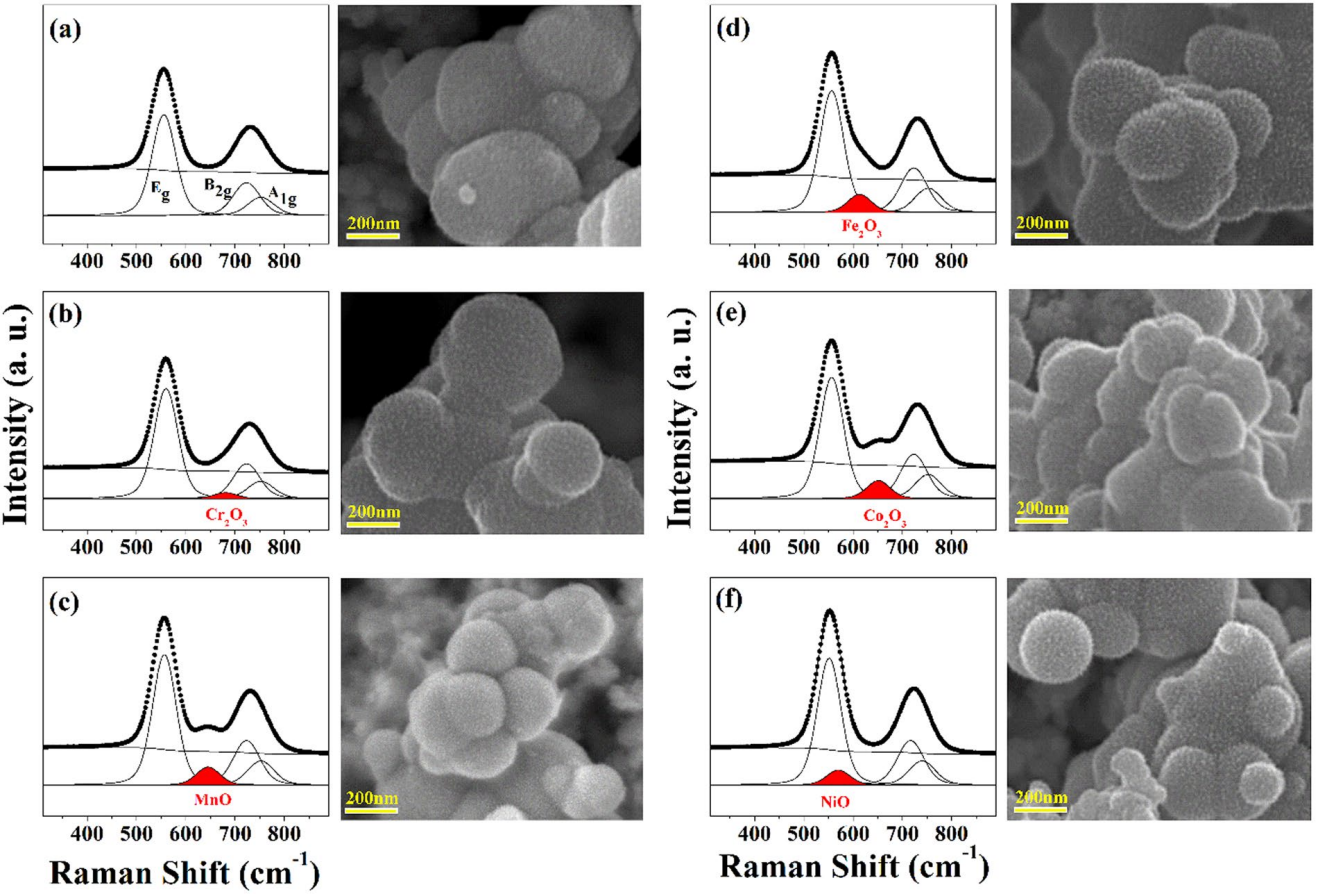
Raman spectra and SEM images of (**a**) IrO_2_, and TM-IrO_2_ for TM = (**b**) Cr, (**c**) Mn, (**d**) Fe, (**e**) Co, and (**f**) Ni.

First, Raman spectra of bare IrO_2_ NPs and the five different TM-IrO_2_ NPs (see Fig. 1) were obtained and the major Raman shifts at ~561 (E_g_), 723 (B_2g_), and 752 cm^−1^ (A_1g_) are well associated with typical rutile IrO_2_ ^25^. Compared with the non-doped IrO_2_ NPs, TM-IrO_2_ NPs was confirmed to have no significant changes in these Raman shifts, except additional minor peaks. These minor peaks are considered as dopant-related signals because they were observed near a Raman shift similar to that of its metal oxide bonds (Cr_2_O_3_: 680, 612, MnO: 644, Fe_2_O_3_: 612, Co_2_O_3_: 651, and NiO: 569 cm^−1^)^26–30^. In addition, SEM images were also obtained to determine the surface morphology of IrO_2_ and TM-IrO_2_ NPs which have mostly similar round partice shapes and small protrusion on the surface. Scanning transmission electron microscope (STEM) images and energy-dispersive X-ray spectroscopy (EDS) mapping images shows Ir and doped transition metal were uniformly distributed over the nanoparticles (Supporting Information Fig. S1).

**Figure 2:**
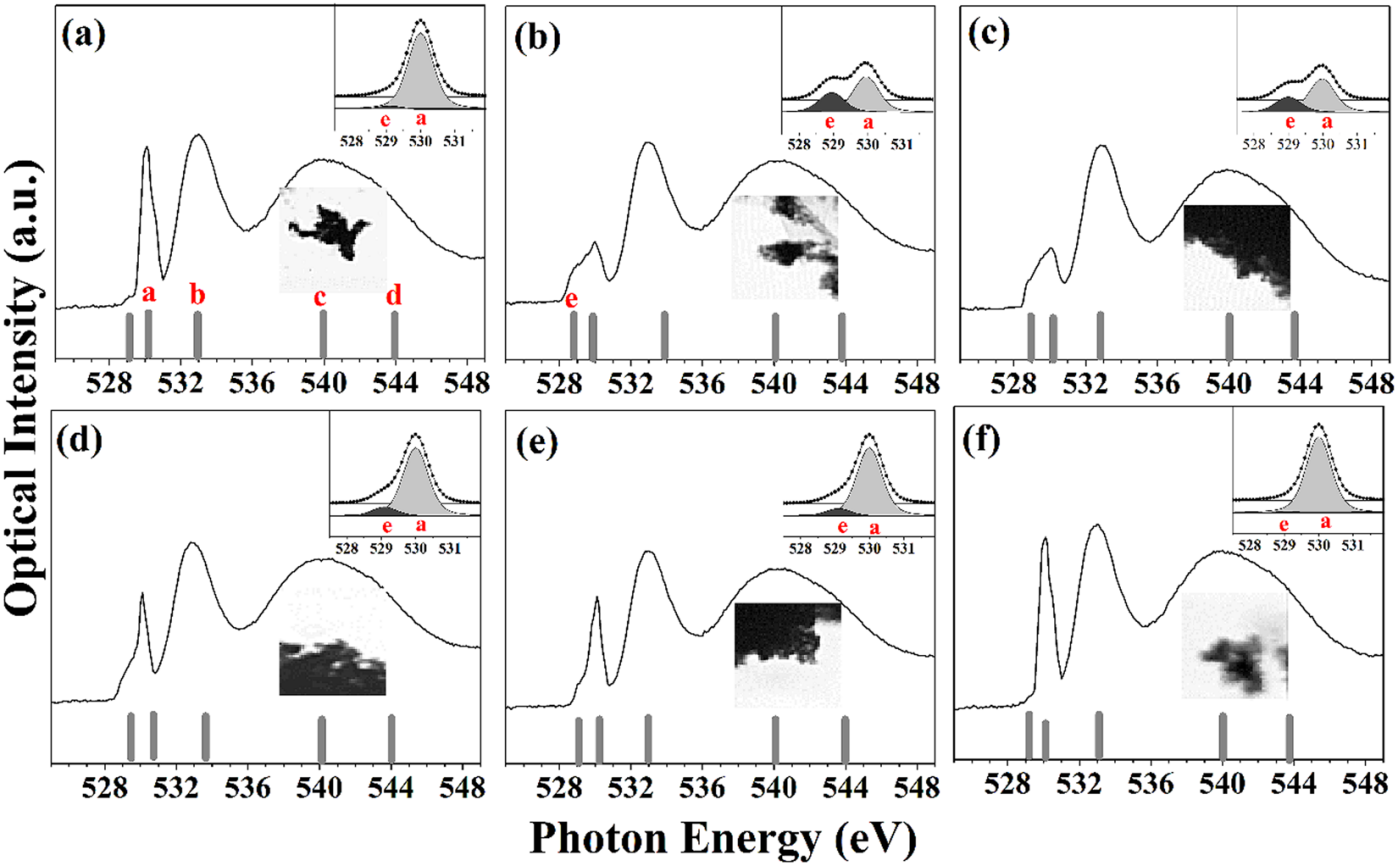
XAS spectra of O *K*-edge and the corresponding stacked images for (**a**) IrO_2_, (**b**) Cr-IrO_2_, (**c**) Mn-IrO_2_, (**d)** Fe-IrO_2_, (**e**) Co-IrO_2_, and (**f**) Ni-IrO_2_ NPs. Inset plots show a magnified view of the spectra at the locations of peaks *e* and *a* after background corrections.

Figure 2 shows the X-ray absorption spectroscopy (XAS) results stacked in STXM images of the IrO_2_ samples, black regions. We focused on the O *K*-edge spectra to characterize the changes of the oxygen states as the result of the doping. Peaks at 530~535 eV are generally observed due to the excitation of electrons from the O 1 *s* state to the O 2*p* –metal *d* hybrid state; peaks at 540~545 eV, were by excitation from the O 1 *s* state to the unoccupied *p* state; and the peak at about 528.8 eV (denoted as *e*) was observed in the formation of an amorphous or defect structure on the surface^31^. Specifically, non-doped bare IrO_2_ NPs have four main peaks at 530.1, 532.9, 539.5, and 544 eV (denoted as a, b, c, and *d*, respectively), which are matched with those of typical rutile IrO_2_ structures^31,32^. Strong and relative sharp absorption at 530 eV and 533 eV were reported to be induced by the Ir^4+^ and O^2−^ in rutile IrO_2_ from a computed O *K*-edge spectrum, as well^31^.

Notably, although all of the TM-IrO_2_ NPs have O *K-*edge features at similar position, peak shape and relative intensity near 530 eV was dramatically modified depending on the types of the dopants. In detail, both of the Cr-IrO_2_ and Mn-IrO_2_ NPs had particularly low intensity of peak *a*, and the broaden peak around 530 eV due to strong appearance of an additional peak (peak *e*) at a photon energy of 529 eV (Fig. 2b,c). These differences with dopants can be understood by alteration of the hybridization between the O 2*p* and metal *d* orbitals. The pre-edge signal at ~529 eV is proposed to attribute to iridium vacancies or O 2*p* hole states by the presence of O^−^ species in rutile IrO_2_ ^31^. The hole states created by Cr or Mn doping would be expected to cause an increase of the resonance at peak *e* and consequent decrease at peak *a*. On the other hand, compared to the non-doped IrO_2_ NP, Fe-IrO_2_ and Co-IrO_2_ NPs show changes near 529~530 eV but much less changes than Cr-IrO_2_ or Mn-IrO_2_ (Fig. 2d,e). In other words, decrease in the intensity of peak *a* was smaller and increase of peak *e* was also smaller in the case of Fe or Co doped ones compared with Cr or Mn doped ones. Lastly, the peak shapes of Ni-IrO_2_ turns to be very similar to those of IrO_2_ NPs. The O *K*-edge spectra revealed more generation of the vacancies states when Cr or Mn was incorporated into IrO_2_. Changes of the spectra was large with the transition metals on the left side of the periodic table and decrease with the order of Fe, Cu, and Ni across the periodic table. Note that the transition metal-oxygen bond formation energy levels are greater for those metals on the left side of the periodic table than for those on the right side, and such stronger bonds may have stabilized the vacancies in our IrO_2_ NPs containing doped Cr or Mn^33^. Therefore, the result of O K-edge spectra can classify the dopants into the following three: (i) Cr and Mn which changes the O states the most, (ii) Fe and Co which changes in a milder amount, (iii) Ni which changes least.

Next, HRPES spectra of TM-IrO_2_ NPs are compared in the O 1 *s* and Ir 4*f* regions (Supporting Information Fig. S2), having peaks centered at 529.7 eV and 61.7 eV, respectively, and changes of the spectra induced by new feature were observed indicating modification of the Ir-O interaction. The deconvolution of the O 1 *s* peaks of all the TM-IrO_2_ NPs yielded two minor peaks: one at ~531.0 eV (denoted as O_v_), which might be associated with the defective oxygen structure in IrO_2_, and the other at ~533 eV (denoted as TM-IrO_x_; TM = Cr, Mn, Fe, Co, or Ni)^34,35^. Similarly, a new set of minor peaks generated in the Ir 4*f* spectra at a higher binding energy state, which may have been attributed to Ir^3+^. The Ir^3+^ state has often been reported to be present in amorphous or anodized IrO_x_ structures. Note that at both O 1 *s* and Ir 4*f* spectra, the intensities of the minor signals (i.e. O_v_ and Ir^3+^) were higher for Cr- or Mn-IrO_2_ NPs than those for the other dopant-containing IrO_2_ NPs, which provided a hint that these features are related to each other. These HRPES spectra indicate quite consistent dependence with those of the O *K*-edge XAS spectra.

### Electrocatalytic OER activity of TM-IrO_2_ NP

**Figure 3:**
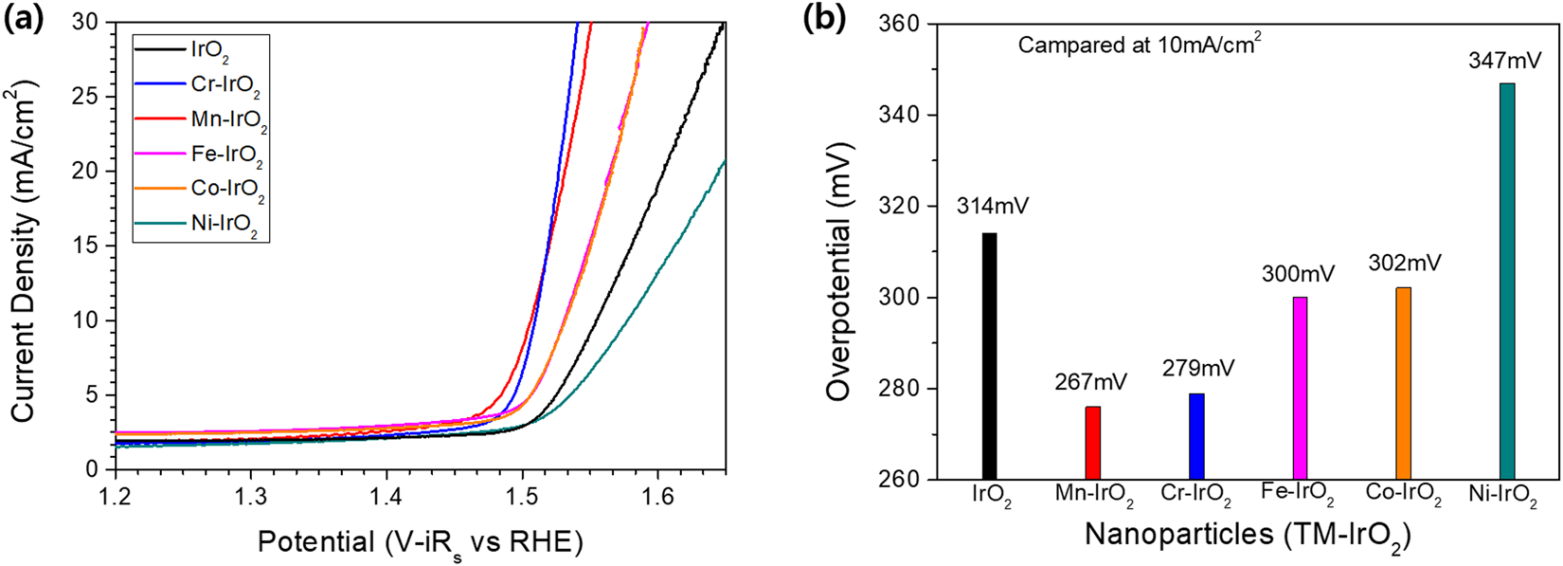
(**a**) Current density-potential curves with iR correction of IrO_2_ and TM-IrO_2_ measured in 1 M KOH, and (**b**) their comparison of overpotential values at 10 mA/cm^2^ current density.

Electrocatalytic OER activities of TM-IrO_2_ NPs were evaluated in the alkaline 1 M KOH electrolyte condition (Fig. 3), exhibiting distinctively different activities. Because the loaded catalyst amounts and the exposed area on the glassy carbon electrode were controlled to be same for the IrO_2_ and TM-IrO_2_ NPs, the trend of the mass activity (mA/mg) normalized by the catalyst loading was the same as that of the current density (mA/cm^2^). Cr-IrO_2_ and Mn-IrO_2_ showed the greatest increases in OER activity, Fe-IrO_2_ and Co-IrO_2_ showed modest increases, and Ni-IrO_2_ showed a rather decrease in activity, compared to the IrO_2_ NPs, (Fig. 3(a)). At a current density of 10 mA/cm^2^, the over-potential (η) values of Cr-IrO_2_ and Mn-IrO_2_ were 267 mV and 279 mV, about 35 mV less than that of the IrO_2_ NPs. It is notable the enhanced overpotentials of these TM-IrO_2_ NPs are also comparable values with those of the high performing OER catalysts^36^ (Supporting Information Table S1). Meanwhile the η of Fe-IrO_2_ and Co-IrO_2_ were about 10 mV less than that of the IrO_2_ NPs. Ni-IrO_2_ required an η value 33 mV greater than that of the IrO_2_ NPs to reach an OER activity of 10 mA/cm^2^ (Fig. 3(b)). Higher OER activities were achieved with the TM-IrO_2_ having more defect-related features according to XAS spectra shown in Fig. 2. Therefore, we propose the vacancy defect states in TM-IrO_2_ NPs to be the active sites displaying the higher electrochemical OER activities.

### Photocatalytic oxidation activity

**Figure 4:**
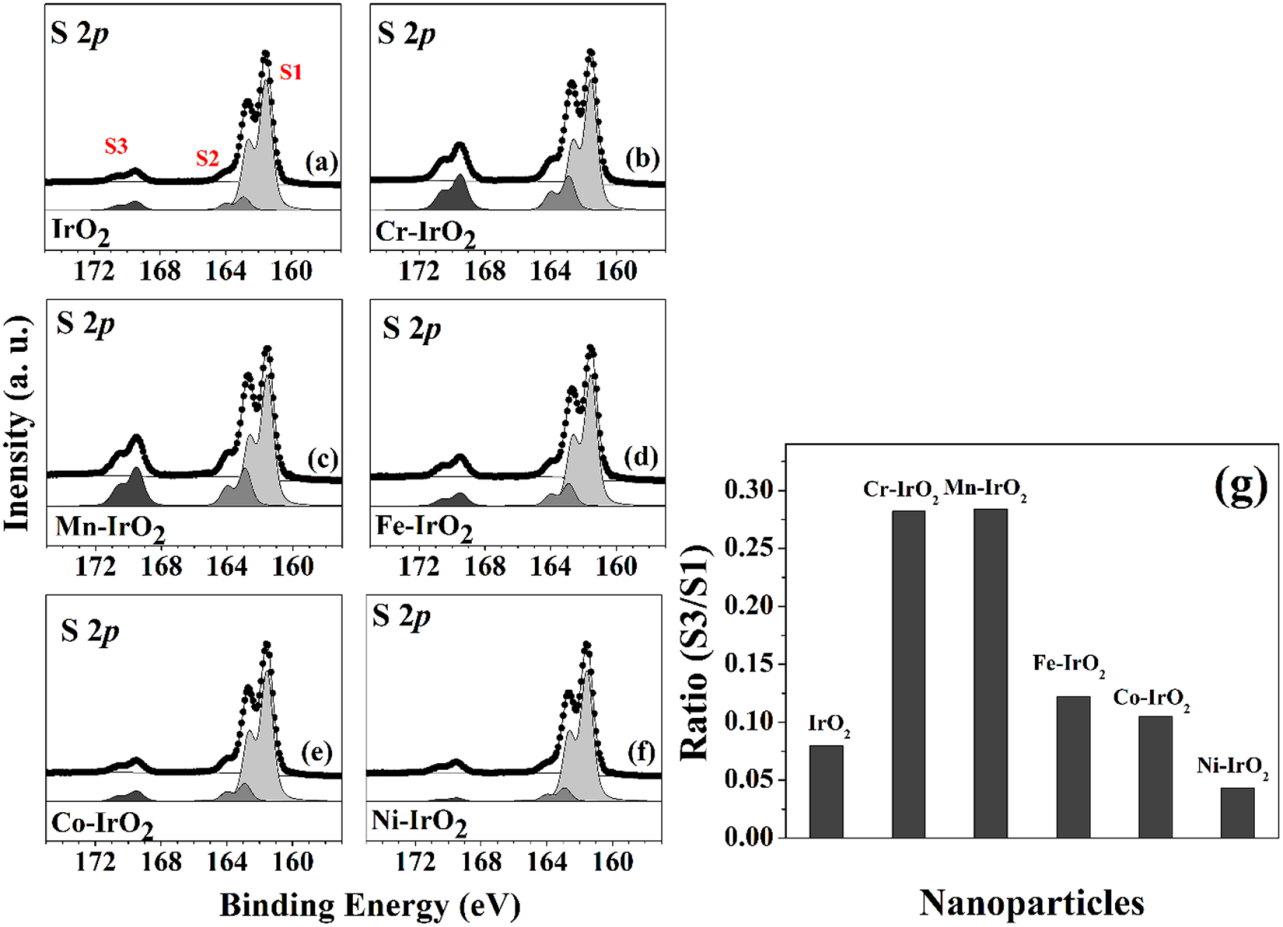
(Left panel, a–f) HRPES S 2*p* core-level spectra obtained after the catalytic oxidations of 180 L 2-ATP (the saturation exposure in our system) on IrO_2_ NPs and 5 mol% TM-IrO_2_ (**a**) IrO_2_, (**b**) Cr-IrO_2_, (**c**) Mn-IrO_2_, (**d**) Fe-IrO_2_, (**e**) Co-IrO_2_, and (**f**) Ni-IrO_2_. (Right panel) (**g**) The ratios of the intensity of the S3 (-SO_3_H) peak to that of the S1 (-SH) peak for the IrO_2_ NPs and the five TM-IrO_2_ samples, indicating their catalytic activities in the oxidation of 2-ATP, for 180 L exposures under 365-nm-wavelength UV light.

We determined the photocatalytic activities of 2-ATP oxidation by surface-sensitive S 2*p* core-level HRPES spectra acquired from the products of the exposure of 180 L of 2-ATP to oxygen and 365-nm-wavelength UV light in the presence of each type of TM-IrO_2_ NPs (Fig. 4(a–f)). Three distinct 2*p*_3/2_ peaks were observed, at 161.5, 162.9, and 168.6 eV, which corresponded to the thiol group (-SH; denoted as S1), the bound state (denoted as S2), and sulfonic acid (SO_3_H) (denoted as S3), respectively. Since sulfonic acid is formed as the result of the oxidation product of the thiol group^37,38^, and the unreacted one is observed at peak S1, we monitored the oxidation of 2-ATP by measuring the ratio of the intensity of S3 to that of S1. These oxidation also showed the same activity trends (Fig. 4(g)). Cr-IrO_2_ or Mn-IrO_2_ exhibited clear enhancements in photocatalytic activity compared with bare IrO_2_, and the conversion ratio decreased in the order of Fe-IrO_2_, Co-IrO_2_, IrO_2_, and Ni-IrO_2_. These results showed closely correlated activity trend between thiol oxidation and OER.

### CO oxidation activity

**Figure 5:**
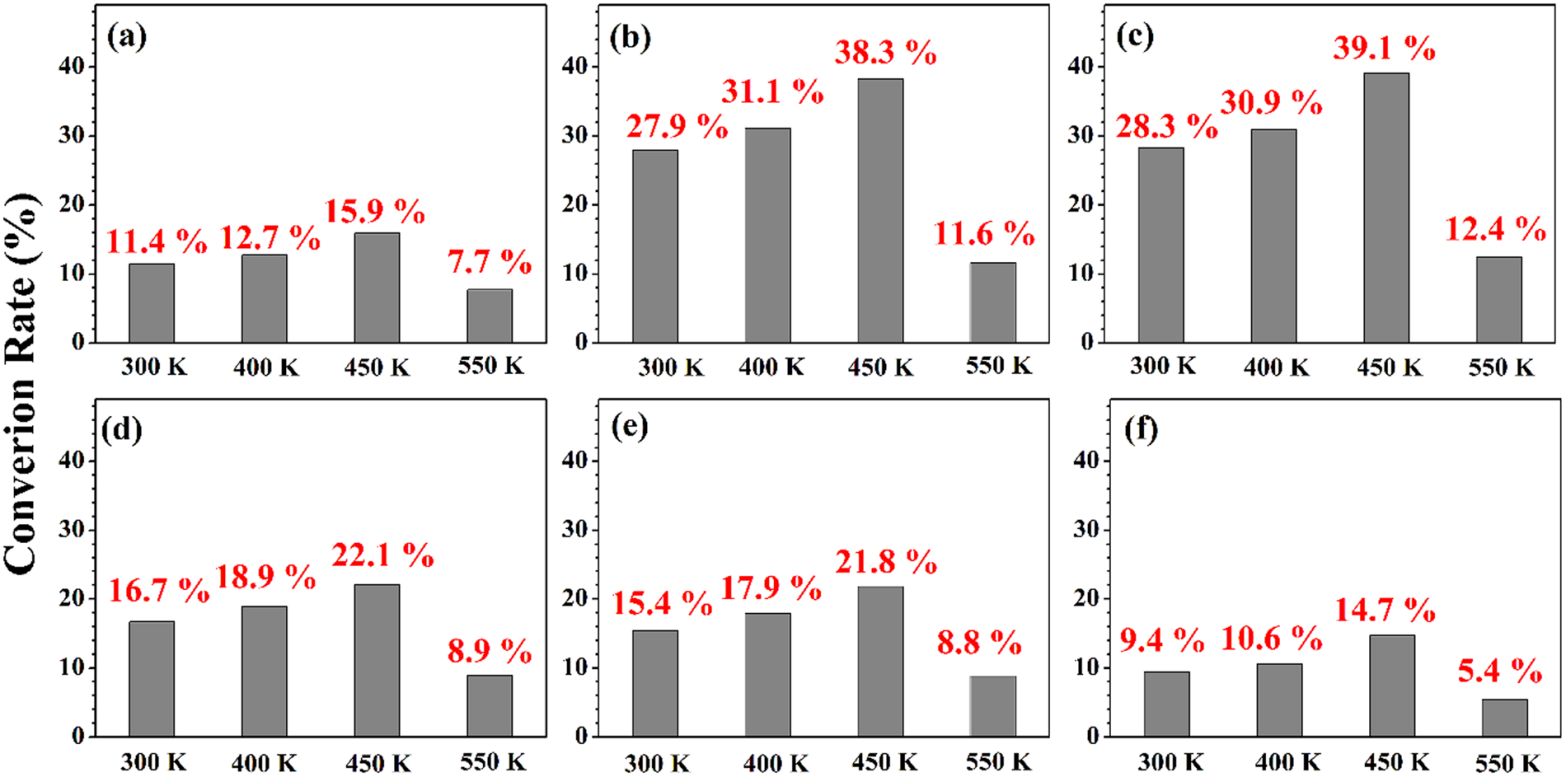
The rates of conversion of CO to CO_2_ gas at various substrate temperatures in the presence of (**a**) IrO_2_, (**b**) Cr- IrO_2_, (**c**) Mn-IrO_2_, (**d**) Fe-IrO_2_, (**e**) Co-IrO_2_, and (**f**) Ni-IrO_2_.

In addition, we assessed the catalytic properties for thermal oxidation conversion of CO to CO_2_. The conversion reactions of each catalyst was monitored at various temperatures between 300 K and 550 K by using mass spectrometry (Fig. 5(a~f)). For all IrO_2_ based catalyst samples, the conversion of CO to CO_2_ steadly increased in the range 300 K to 450 K but rather decreased above 450 K probably due to desorption of the reactant chemicals on the catalyst surface before conversion. When we compare the CO oxidative conversion activity of the six catalysts at 450 K, again Cr- or Mn-doped IrO_2_ NPs showed much enhanced converaion rate, and the conversion rates follow the same order at other reaction temperature as well. Overall, CO to CO_2_ conversion using TM-IrO_2_ catalysts exhibits the same order of the activity as electrochemical OER or photocatalytic 2-ATP oxidation.

### Common oxidation activity trends

By characterizing oxidation activites related to the electronic structures of bare IrO_2_ and five different TM-IrO_2_ NP catalysts, we found that TM-IrO_2_ catalysts had the same activity trends for three quite different reaction conditions (i.e. electrochemical oxidation of water in an aqueous condition, photocatalytic oxidation of 2-ATP in ultra-high vacuum condition, and thermal CO oxidation. For all oxidation reactions, five different TM-IrO_2_ catalysts can be roughly divided into three groups in terms of the activity: (i) Cr-IrO_2_ and Mn-IrO_2_ which had similarly high enhancement in activity, (ii) Fe-IrO_2_ and Co-IrO_2_ which had less enhancement, and (iii) Ni-IrO_2_ which had similar activity compared with non-doped IrO_2_. These results indicate there can be a common main contributor to catalytic activity, which is independent on the specific environmental conditions of each reation. In other words, changes in the electrical conductivity might appear important to determine electrocatalytic OER reaction activity but they are not expected to be critical for thermal CO oxidation. Or, doping in a photocatalyst material has been demonstrated to be effective to vary bandgaps or band positions affecting the photocatalytic acitivty, but it would not matter directly to electrochemical or thermal catalytic activity.

Based on the spectroscopic surface analysis studies, a reasonable explanation is that the dopant metals disturb the Ir-O interaction and create oxygen vacancy defective states which are crucial to determine the catalytic activity of various oxidation reaciton. It was observed that electronic states of TM-IrO_2_ were modified especially in the O K-edge XAS signal related with O *2p* and Ir *4d* orbital interaction. A higher proportion of defect states appeared in Cr-IrO_2_ and Mn-IrO_2_ and decreased with the transition metal dopant in the order of Fe, Co, and Ni across the periodic table. Interestingly, the electronegativity values of Cr and Mn are close to each other as 1.66 and 1.55, respectively, and those of Fe and Co are similar as 1.83 and 1.88, respectivley, while Ni is slightly larger, 1.91. All of these values are smaller than the electronegativity value of Ir (=2.2). This electronegativity hypothesis and the lower level of hybridization between the doped metal *d* and O 2*p* orbitals may explain our results in a fairly straightforward and reasonable manner, but this explanation requires further studies to be proven.

## Conclusion

In this study, transition metal doped IrO_2_ nanoparticles were prepared and their catalytic activities were compared for three different types of oxidation reactions such as electrochemical oxygen evolution reaction, photocatalytic 2-ATP thiol oxidation reaction, and thermal CO conversion to CO_2_. Interestingly, Cr-IrO_2_ or Mn-IrO_2_ showed the most superior catalytic activity for all oxidation reactions followed by Fe-IrO_2_ and Co-IrO_2_, and Ni-IrO_2_ had similar or even decreased activity compared with non-doped IrO_2_ NPs. In other words, these five TM-IrO_2_ catalysts were classified into three group in terms of enhancement of the catalytic activity. For instance, the Cr- or Mn-IrO_2_ exhibited decreased OER overpotential at 10 mA/cm^2^ by 35~30 mV compared with non-doped IrO_2_, while the overpotential of Ni-IrO_2_ rather increased by ~30 mV. These activity trends were also in good agreement with our STXM and HRPES measurements that showing Cr-IrO_2_ and Mn-IrO_2_ had the most changes in O *K*-edge or Ir *4f* spectra related to the oxygen vacancy sites of IrO_2_. Meanwhile, Fe- or Co-IrO_2_ had mild, and Ni-IrO_2_ had smallest evolution of defect-related features compared with IrO_2_. These combined experimental results suggest that, among five transition metals, Cr- or Mn- dopant in IrO_2_ are the most effective to generate defect structures by modifying the electronic/chemical states and thus contribute to enhanced activity for various oxidation reactions.

## Methods

### Preparation of IrO_2_ and TM-IrO_2_ nanoparticles

Potassium hexachloroiridate (K_2_IrCl_6_) and all dopant precursors (such as Cr(NO_3_)_3_∙9H_2_O, Mn(NO_3_)_2_∙xH_2_O, Fe(NO_3_)_3_∙9H_2_O, Co(NO_3_)_3_∙9H_2_O, and Ni(NO_3_)_2_∙6H_2_O) were purchased from Sigma-Aldrich, and sodium hydroxide (NaOH) was obtained from Samchun. All aqueous solutions were prepared using double distilled water (DDW).

The IrO_2_ NP solution was prepared by modifying Wohler’s method^39^. K_2_IrCl_6_ (120 mg, 0.25 mmol) was dissolved in water in a flask to produce 100 mL of the corresponding aqueous solution, to which 1 mL of an aqueous NaOH solution (25 wt%) was added. The flask was capped with a foil, the solution in the flask mixed, and immediately thereafter the foil cap was taken off and the flask was placed in a 90 °C oil bath for 20 min. The resultant solution was subsequently cooled to room temperature in an ice bath and stored in a capped vial for more than 24 hours. Upon being heated, the initially dark green solution became increasingly transparent, but then dark blue at 90 °C, as desired. An increasing quantity of particles were seen over time after the heating was completed. After being stored at room temperature, the upper layers of the heated solution separated from the upper level of the upper stream, and sediment eventually formed over this separation. But when heated to 90 °C, no such sediment was found.

To prepare 5 wt% doped TM-IrO_2_ with different metal contents, the desired amount of dopant (TM(NO_3_)_x_∙*n*H_2_O solution) was determined in each case by calculation of mole fraction and then added into a solution of IrO_2_ NPs under continuous stirring. The mixed solution was transferred to a Teflon-lined autoclave, then placed in a convection oven preheated at 220 °C for seven hours. The product was washed and collected by precipitation several times with copious amounts of DDW.

### Material characterizations

Raman spectra were obtained by using a spectrometer (Horiba, ARAMIS) with an Ar^+^ ion CW (514.5-nm-wavelength) laser. The morphologies of the samples were characterized by using a field-emission scanning electron microscope (FE-SEM, FEI Inspect F50) operating at an acceleration voltage of 10 kV. Element distributions of Ir and dopant metal were characterized by using a scanning transmission microscope, energy-dispersive X-ray spectroscopy (STEM-EDS, FEI Talos F200X). A scanning transmission X-ray microscopy (STXM) was performed at the 10A beamline of the Pohang Accelerator Laboratory (PAL). A Fresnel zone plate with an outermost zone width of 25 nm was used to focus the X-rays onto the TM-IrO_2_ on the TEM grids. Image stacks were acquired using X-ray absorption spectroscopy (XAS) to extract the O *K*-edge spectra. High-resolution photoemission spectroscopy (HRPES) experiments were performed at the 8A1 beamline of the PAL with an electron analyzer (Physical Electronics, PHI-3057). The binding energies of the core level spectra were determined with respect to the binding energy (E_B_ = 84.0 eV) of the clean Au 4 *f* core level for the same photon energy.

### Electrocatalytic OER performance

The electrocatalytic performances of the prepared nanoparticles were compared for the OER in 1 M KOH electrolyte. Each IrO_2_ NP and TM-IrO_2_ sample (10 mg) was dispersed in a solution containing Nafion (100 *μ*L; 5wt %, DuPount) and either absolute ethanol (2 mL; Daejung, 99.9%) or 2-propanol (2 mL; Samchun, 99.5%) by ultra-sonicating the mixture for 30 min. The prepared IrO_2_ NP or TM-IrO_2_ catalyst ink samples were drop-cast on a clean glassy carbon electrode (Alfa Aesar), and the area density of the loaded nanoparticle catalyst was controlled to be 2.8 ± 0.4 mg/cm^2^. The catalyst nanoparticles deposited on the glassy carbon electrodes were masked to have an exposed area of 0.5 cm^2^. The electrochemical OER activities were measured in 1 M KOH (Aldrich, 85% purity), pH 13.7, by carrying out cyclic voltammetry at a scan rate of 10 mV/sec using a potentiostat (Ivium Technologies). A coiled Pt wire and an Hg/HgO (1 M NaOH, CH Instruments) electrode were used as a counter and reference electrode, respectively. All of the measured potentials were converted to being versus a reversible hydrogen electrode (RHE) using the equation

1 $${{\boldsymbol{E}}}_{{\boldsymbol{RHE}}}={{\boldsymbol{E}}}_{{\boldsymbol{Hg}}/{\boldsymbol{HgO}}}+{\bf{0.059}}\,{\boldsymbol{V}}\times {\boldsymbol{pH}}+{{\boldsymbol{E}}}_{{\boldsymbol{Hg}}/{\boldsymbol{HgO}}}^{{\bf{0}}},\,{{\boldsymbol{E}}}_{{\boldsymbol{Hg}}/{\boldsymbol{HgO}}}^{{\bf{0}}}={\bf{0}}.\,{\bf{140}}\,{\boldsymbol{V}}$$

To compensate for the potential drop resulting from solution resistance (R_s_), electrochemical impedance spectra (EIS) were acquired in the frequency range 100 kHz – 0.1 Hz, and a typical R_s_ of ~5–10 Ω was obtained in 1 M KOH electrolyte.

### Photocatalytic oxidation performance

2-Aminothio-phenol (C_4_H_4_SHNH_2_, 2-ATP) was purified by carrying out turbo pumping prior to dosing it onto the IrO_2_ NPs and the five TM-IrO_2_ samples. The dosing was carried out by using a direct dozer controlled by means of a variable leak valve. The resulting samples were then irradiated with UV light (λ = 365 nm, VL-4.LC. Tube 1 × 4-Watt, Vilber Lourmat) through a quartz window of a vacuum chamber. The pressure of the chamber was maintained at 10^−6^ Torr during dosing, and the number of exposed molecules was defined by the dosing time in seconds, i.e., with one Langmuir (L) corresponding to one second of dosing under 10^−6^ Torr.

### Thermal CO oxidation

The synthesized catalyst was dispersed in an aqueous solution (0.05 mg/10 mL) which was spin-coated to load on a copper substrate. Then, the catalyst loaded substrate was transferred into the ultra high vacuum (UHV) chamber for CO oxidation reaction. CO gas was flew into the chamber until the partial pressure became 10^−6^ torr, and the same amount of O_2_ gas was added. The catalyst was heated up to reaction temperature (300, 400, 450, or 550 K), and UV lamp was turned on through the view-port of UHV chamber to activate O_2_. The CO oxidation reaction was conducted for 1 hour and the amount of the generated CO_2_ was measured by a Hiden RC 301 mass spectroscopy (mass range ~300 amu). The reaction was repeated by five times at each condition and the average conversion rate was taken.

## Electronic supplementary material

Supplementary files
