# Supplementary material for: Comparative study of catalytic activities among transition metal-doped IrO2 nanoparticles
Source: Sci Rep. 2018 Nov 13;8:16777. doi: 10.1038/s41598-018-35116-w (PMC6233218; doi:10.1038/s41598-018-35116-w)
Supplement: Supplementary file 1 — Supplementary files [file 41598_2018_35116_MOESM1_ESM.docx]

Supporting information

**Comparative study of catalytic activities among transition metal-doped IrO_2_ nanoparticles**

Hangil Lee,^1^ Joo Yeon Kim,^1^ Si Young Lee,^2,3^ Jung A Hong,^1^ Namdong Kim,^4^ Jae Yoon Baik,^4^ and Yun Jeong Hwang^*2,3^

*^1^* Department of Chemistry, Sookmyung Women's University, Seoul 140-742, Republic of Korea

*^2^* Clean Energy Research Center, Korea Institute of Science and Technology, Seoul 02792, Republic of Korea

*^3^* Division of Energy and Environmental Technology, KIST School, Korea University of Science and Technology, Seoul 02792, Republic of Korea

*^4^* Beamline Research Division, Pohang Accelerator Laboratory (PAL), Pohang 790-784, Republic of Korea.

Corresponding Author

*Yun Jeong Hwang Tel.: +82 2 958 5227; fax: +82 2 958 5809 E-mail: yjhwang@kist.re.kr


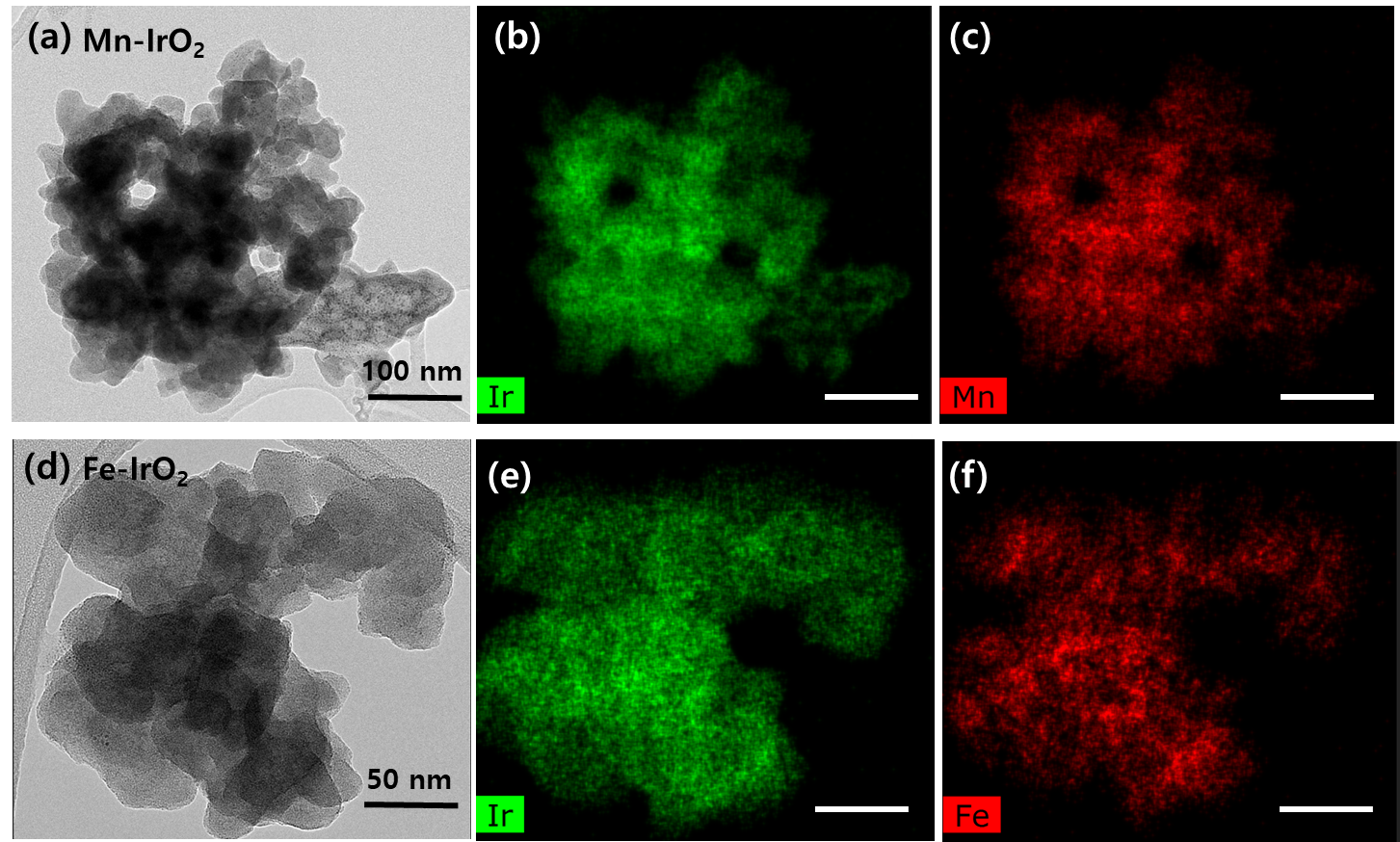


**Figure S1**. (a-c) TEM and EDS mapping images of Mn-IrO_2_, and (d-f) TEM and EDS mapping images of Mn-IrO_2_


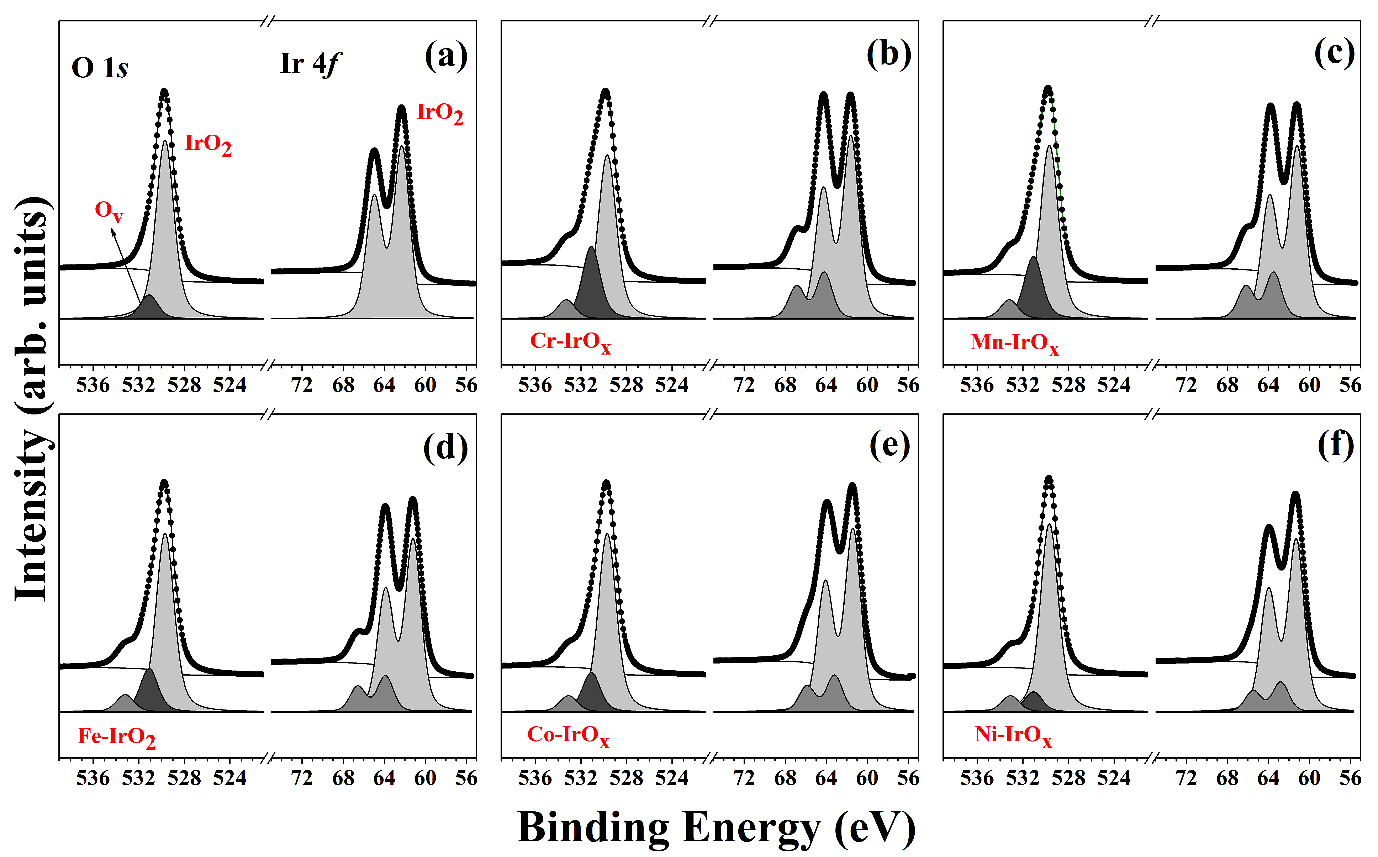


Figure S2. The O 1*s* and Ir 4*f* regions of the HRPES spectra of (a) IrO_2_, (b) Cr-IrO_2_, (c) Mn-IrO_2_, (d) Fe-IrO_2_, (e) Co-IrO_2_, and (f) Ni-IrO_2_ NPs. O_v_ indicates to oxygen vacancy induced peak.

**Table S1.** Comparison of OER activity with metal or metal oxide catalysts

**
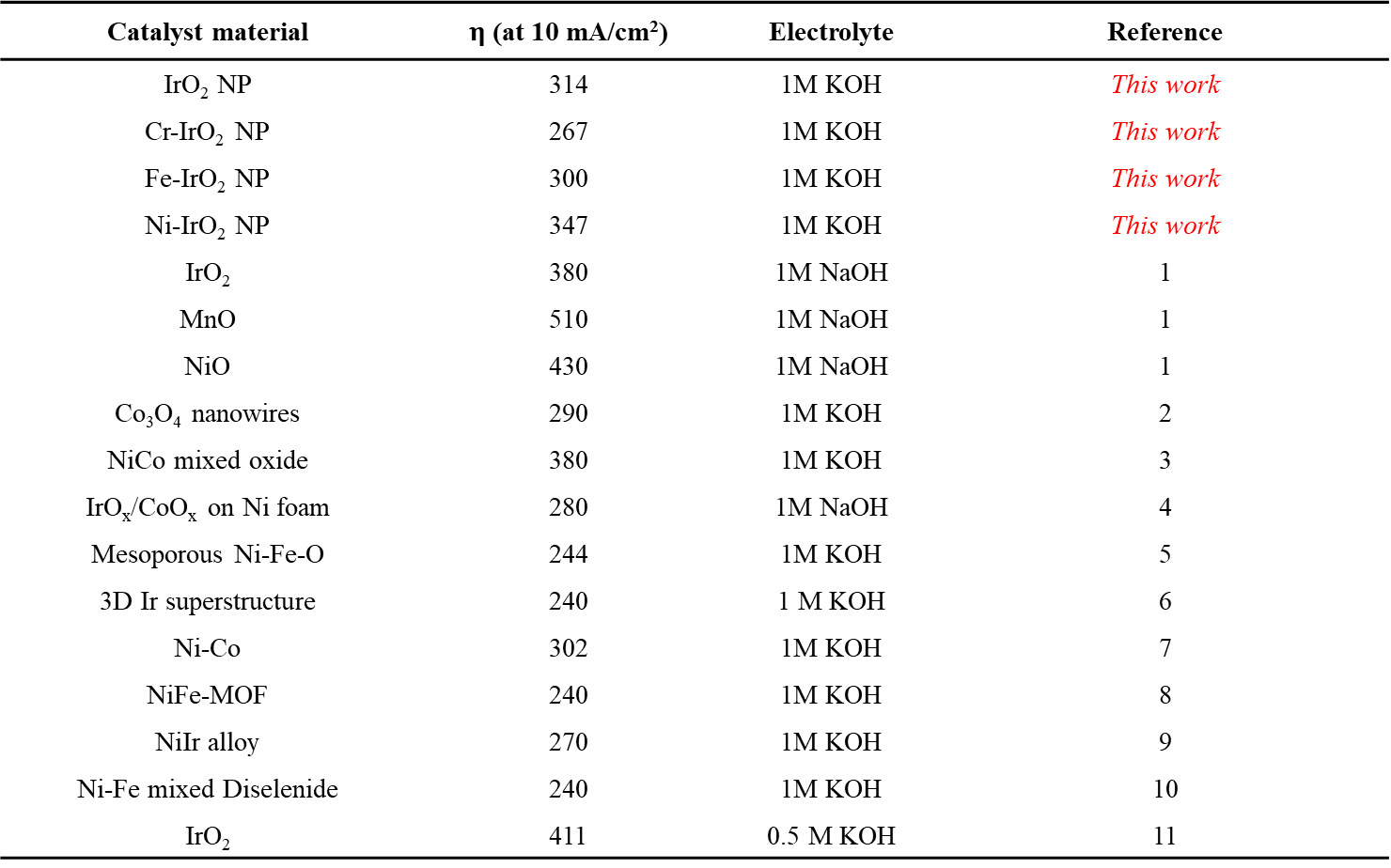
**

**References**

1. Jung, S., McCrory, C., Ferrer, I., Peters, J., Jaramillo, T., Benchmarking nanoparticulate metal oxide electrocatalysts for the alkaline water oxidation reaction, *J. Mater. Chem. A* **4**, 3068-3076 (2016).
2. Wang, Y., Zhou, T., Jiang, K., Da, P., Peng, Z., Tang, J., Kong, B., Cai, W., Yang, Z., Zheng, G. Reduced Mesorporous Co_3_O_4_ Nanowires as Efficient Water Oxidation Electrocatalysts and Supercapacitor Electrodes, *Adv. Energy Mater.* **4**, 1400696 (2014).
3. Nardi, K., Yang, N., Dickens, C., Strickler, A., Bent, S. Creating Highly Active Atomic Layer Deposited NiO Electrocatalysts for the Oxygen Evolution Reaction, *Adv. Energy Mater.* **5**, 1500412 (2015).
4. Tae, E., Song, J., Lee, A., Kim, C., Yoon, S., Hwang, I., Kim, M., Yoon, K. Cobalt Oxide Electrode Doped with Iridium Oxide as Highly Efficient Water Oxidation Electrode, *ACS Catal.* **5**, 5525-5529 (2015).
5. Dong, C., Kou, T., Gao, H., Peng, Z., Zhang, Z. Eutectic-Derived mesoporous Ni-Fe-O nanowire network catalyzing oxygen evolution and overall water splitting, *Adv. Energy Mater.* **8**, 1701347 (2018).
6. Pi, Y., Zhang, N., Guo, S., Guo, J., Huang, X. Ultrathin Laminar Ir Superstructure as Highly Efficient Oxygen Evolution Electrocatalyst in Broad pH Range, *Nano Lett.* **16**, 4424-4430 (2016).
7. Bae, S., Kim, J., Randriamahazaka, H., Moon, S., Park, J., Oh, I., Seamlessly Conductive 3D Nanoarchitecture of Core-Shell Ni-Co Nanowire Network for Highly Efficient Oxygen Evolution, *Adv. Energy Mater.* **7**, 1601492 (2016).
8. Duan, J., Chen, S., Zhao, C.,Ultrathin metal-organic framework array for efficient electrocatalytic water splitting, *Nat. Comm.* **8**, 15341 (2017).
9. Pi, Y., Shao, Q., Wang, P., Guo, J., Huang, X. General Formation of Monodisperse IrM (M = Ni, Co, Fe) Bimetallic Nanoclusters as Bifunctional Electrocatalysts for Acidic Overall Water Splitting. *Adv. Funct. Mater.* **27**, 1700886 (2017).
10. Nai, J., Lu, Y., Yu, L., Wang, X., Lou, X. Formaiton of Ni-Fe Mixed Diselenide Nanocages as a Superior Oxygen Evolution Electrocatalyst *Adv. Mater.* **29**, 1703870 (2017).
11. Suen, N. –T., Hung, S. –F., Quan, Q., Zhang, N., Xu, Y. –J., Chen, H. M., Electrocatalysis for the oxygen evolution reaction: recent development and future perspective, *Chem. Soc. Rev.* **46**, 337-365 (2017).
